# Impact of COVID-19 on Utilization of Healthcare Services Among People Living with HIV (PLHIV): A Systematic Review

**DOI:** 10.3390/medicina61010111

**Published:** 2025-01-14

**Authors:** Enyinnaya Ukaegbu, Tilektes Maulenkul, Antonio Sarria-Santamera

**Affiliations:** 1 Department of Biomedical Sciences, School of Medicine, Nazarbayev University, Kerey and Zhanibek Khans St. 5/1, Astana 010000, Kazakhstan; 2 Fortitude Valley Campus, Torrens University, 90 Bowen Tce, Brisbane, QLD 4006, Australia; tilektes.maulenkul@ayu.edu.kz; 3 Science Department, Khoja Akhmet Yassawi Kazakh-Turkish International University, Turkistan 161200, Kazakhstan

**Keywords:** COVID-19 impacts, SARS-CoV-2, HIV/AIDS, health service utilization, women’s health

## Abstract

*Background and Objectives:* People living with HIV/AIDS have been impacted notably by the COVID-19 pandemic in diverse dimensions. Although some impacts of COVID-19 on PLHIV have been well documented, there is still insufficient research that captures the overall understanding of the implications of COVID-19 for the healthcare utilization among PLHIV. This review aims to evaluate the impact of COVID-19 on PLHIV, narrowing it down to women living with HIV/AIDS. *Materials and Methods:* An electronic database search of primary studies that obtained information from health facility data regarding PLHIV since 2020 was carried out according to the PRISMA statement. A combination of search terms, e.g., “COVID-19 pandemic”, “SARS-CoV-2”, “Health services utilization”, “People living with Human immunodeficiency virus”, was included. *Results:* There was an overall decline in the utilization of health services such as medical consultations, ART uptake, and viral load monitoring by PLHIV at primary health centers at the forefront of care delivery. However, the countries in the sub-Saharan African region showed a progressive service increase over the pandemic. There was a scarcity of research on the impact of COVID-19 on women populations living with HIV/AIDS. Other methods of healthcare delivery such as telemedicine and postage services were instrumental to the delivery of HIV care services. *Conclusions:* The COVID-19 pandemic reduced the overall uptake of healthcare services among PLHIV and women living with HIV/AIDS. There is a need to boost research and strengthen health emergency preparedness for PLHIV, particularly women living with HIV/AIDS, during pandemics and further harness and maximize the use of contemporary healthcare delivery methods other than the traditional ones.

## 1. Introduction

Severe acute respiratory syndrome coronavirus-2 (SARS-CoV-2), popularly referred to as COVID-19, was first detected in China toward the end of 2019 [1]. It posed a serious threat to several countries and populations, with over 776 million cases and 7.1 million deaths, globally [2]. In March 2020, with the imminent public health risks linked to COVID-19, the World Health Organization (WHO) announced that COVID-19 was an international health emergency requiring urgent response across nations [3]. To mitigate the spread of the disease, the WHO introduced social distancing measures and advised countries to regulate human movement to decrease the rate of transmission of the disease [4]. This restriction of movement “lockdown” greatly impacted every facet of human life, including agriculture, commerce, education, and healthcare [5]. Healthcare services were severely affected by the COVID-19 pandemic, leading to the overall reduction in the activities of care services, admission rates, and low utilization rates of care [6].

Populations such as people living with human immunodeficiency virus (PLHIV) and acquired immunodeficiency syndrome (AIDS) have been influenced by the COVID-19 pandemic and the restriction of movement. The lockdown remarkably hampered access to care services [7,8], a phenomenon which led to the disruption of health services, resulting in fluctuations in routine medical visits, anti-retroviral therapy (ART) uptake, and viral load testing, all of which have been well documented [9,10]. These disruptions in the medical services undoubtedly impacted the healthcare service utilization by PLHIV at different levels, with an associated decrease in medical visits, routine laboratory appointments, and a general lack of access to social care [11,12]. These challenges in seeking care services during the pandemic, marked by anxiety and fear of contracting the COVID-19 infection, presented a problem for PLHIV in terms of coping with the syndemic, thereby creating a double tragedy for the population [13,14]. AIDS-related deaths were estimated to have heightened during the pandemic, with a significant loss in years of life and premature mortality due to COVID-19 [15]. Therefore, it is imperative to holistically identify and evaluate the extent of change in healthcare utilization among PLHIV during the pandemic. This will provide the background for understanding the level of healthcare preparedness for PLHIV during the COVID-19 pandemic and enable proper planning in anticipation of future health emergencies.

Similarly to the HIV infection, there exist gender differences in COVID-19 infections, as women seem to be much more vulnerable to COVID-19 infection than men [16,17]. Hence, the women population represents a fundamental demographic for research with regard to the challenges of healthcare uptake during the COVID-19 pandemic. Although previous research has evaluated the influence of COVID-19 on women’s health [18,19], particularly during the gestational period when women are at a greater risk of exposure [20], there remains limited knowledge regarding women living with HIV (WLHIV) during the pandemic. Owing to this gap in knowledge, it has become imperative to investigate the uptake of health services among WLHIV during the pandemic as they are prone to varying degrees of gender inequities [21]. Therefore, our study aims to identify the impact of the COVID-19 pandemic on healthcare service utilization among PLHIV and to understand the reasons for the amount of use of healthcare services, narrowing it down to women living with HIV (WLHIV).

## 2. Materials and Methods

The study reports this review following the Preferred Reporting Items for Systematic Reviews and Meta-Analysis guidelines (PRISMA) [22]. The systematic review was registered on PROSPERO (CRD42024563377). The research question was ‘What is the impact of COVID-19 on the healthcare utilization of people living with HIV?’

### 2.1. Search Strategy

Due to the variety and number of research articles in the area of COVID-19 and the overlap of studies in search engines, with a unanimous decision, the reviewers decided to search for articles in Scopus and Web of Science only. The search was carried out between 25 July and 16 August 2024. Using a combined search strategy of COVID-19 pandemic OR SARS-CoV-2 AND health service utilization AND people living with human immunodeficiency virus, the researchers were able to curate all relevant literature within the search databases. The combined search is seen in Supplementary Table S1.

### 2.2. Eligibility Criteria

The included articles were:

1. All primary peer-reviewed research that describes a direct link between health service utilization among PLHIV and the COVID-19 pandemic since March 2020, since the declaration of the pandemic by the WHO [3].
2. Studies that collected the data from healthcare centers, HIV service delivery centers, and data from investment programs on HIV/AIDS.
3. Articles that describe the healthcare utilization using a quantitative approach.
4. Studies that state clear aims, objectives, and clearly explain the choice behind the study method/design, with the analysis of the data clearly linked with the study definitions.

Exclusion criteria were: (i) all articles with data collected through an interviewer-administered questionnaire (online or face-to-face), (ii) studies relating to the impact of COVID-19 that are specific to any other vulnerable population groups (such as MSM, PWID, opioid-dependent, and people with STI), (iii) studies relating to perceptions and experiences relating to HIV care services, (iv) studies relating to characteristics, outcomes, and severity of COVID-19 infection among PLHIV, and (v) studies relating to the evaluation of intervention programs. Other reasons for exclusion included papers relating to HIV and COVID-19 that did not meet the inclusion criteria and did not address the research question. Papers such as vaccination assessments and adherence evaluations of PLHIV, general HIV testing services during the pandemic period, responses from healthcare workers on HIV services and pandemic, impact related to pandemic and healthcare services on other populations not related to PLHIV, with the impact not related to HIV including behavioral or psychological issues relating to PLHIV during the pandemic. Case reports, conference papers, dissertations, or personal opinions were also excluded.

### 2.3. Study Extraction

The data were extracted using Microsoft Excel, so that all methodological information from the studies could be compiled and accessible at a glance. Some of the information extracted included the authors, year, country of origin, aim of the study, and key findings.

The main intended outcome of the study is to quantify the use of healthcare services among PLHIV (we identified studies that involved women aged 15 years and above) who were clinically diagnosed with HIV before COVID-19 and were receiving HIV-related services during the COVID-19 pandemic. The secondary outcome is to identify and understand the reasons behind the level of use of healthcare services during the COVID-19 pandemic among PLHIV. T.M. and E.U. conducted the literature search from databases through screening, title review, and duplicate removal. After excluding duplicate articles, abstracts and full texts were independently reviewed by E.U. and T.M. Differences were resolved by a unanimous decision involving the third reviewer A.S.-S.

### 2.4. Quality Appraisal

The quality appraisal of the selected studies was undertaken using checklists based on the type of study to be appraised. Quality assessment tools for cross-sectional and cohort studies were employed for the quality appraisal based on the applicable criteria using the National Heart, Lung, and Blood Study Quality Assessment Tools [23], as seen in Supplementary Table S2. To assess the credibility and integrity of the quantitative studies, Coughlan, Cronin, and Ryan’s framework [24] was used to establish the trustworthiness of studies.

### 2.5. Data Synthesis

To enable the description of the results, our study adopted a narrative synthesis [25]. Patterns were described from the quantitative data that were extracted and grouped into themes: client medical visits, anti-retroviral therapy (ART) initiations, and viral load monitoring. Studies are described and presented in a tabular format. To reduce subjectivity, the third reviewer suggested several outcome measures to be extracted. Due to varying methodologies for describing the utilization of healthcare by PLHIV, the statistical heterogeneity of outcome measures, and the variations in settings in the included studies, a formal meta-analysis was not applicable in this systematic review.

## 3. Results

**Figure 1:**
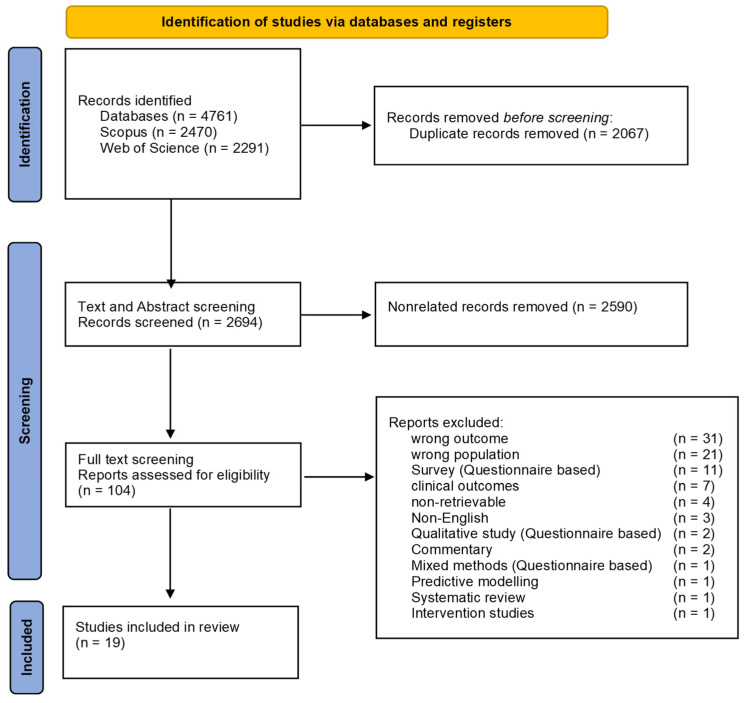
The preferred reporting items for systematic reviews and meta-analysis guidelines (PRISMA) flow diagram.

The studies showed the impact of COVID-19 on PLHIV using data obtained from health facility records. A total of 4761 studies were retrieved from the database search of published primary research studies between 2000 and 2024. A total of 2067 duplicates were removed. A total of 2590 unrelated studies were removed, leaving 104 studies for screening. Most of the studies excluded reported the wrong outcomes and, majorly, discussed HIV testing services during the COVID-19 pandemic for other key populations aside from PLHIV. The following studies [26,27] were excluded because the participants’ age was lower than 15 years. A total of 19 studies were included for review, as seen in the PRISMA diagram below (Figure 1).

### 3.1. Study Characteristics

Out of the 19 studies under review, four were carried out in the Americas—three from North America, United States [28,29,30], and one from the Caribbean region, Haiti [31]—three from the European region—two from Italy [32,33] and one from Spain [34]—one from south-east Asia, Myanmar [35], one from the west Pacific region, Australia [36], and one cross-continental study [37]. The remaining ten studies were from the African region, particularly the sub-Saharan African countries, as seen in the table below (Table 1). Ten studies had a cross-sectional study design, five were cohort studies, and the other four studies utilized retrospective study designs. Among all the studies, only Chappell E. et al. (2023) [38] described health services uptake using women living with HIV as the main population of the study.

Two studies [39,40] evaluated PLHIV with categorized age groups; however, only information on those older than 15 years was extracted for the review. Most studies from sub-Saharan African countries and the study from south-east Asia utilized data retrieved from activities of investment programs. A study [41] used a mixed study to explore the HIV care program in 19 African countries from which only the quantitative data were extracted. Only analyzed data from health facilities were extracted from [31,34,42] studies, and were utilized for the review. Interrupted time series estimations of the studies were excluded from the review because we aimed to understand the real-world influence of the pandemic on PLHIV rather than predictions. Similarly, only analyzed health facility data were used for the review from the study of [43], as regression discontinuity estimations were left out from the review. All the studies explored population data of PLHIV. Only information regarding the PLHIV was retrieved from these studies [33,35,37,39,42] which incorporated other population groups. 

### 3.2. Synthesis

The following will be the headings for the review:

1. **Client medical visits**
  - General population living with HIV

Overall, there were lower rates of in-person medical visits in 2020 during the COVID-19 pandemic, especially during the restriction of movement ‘lockdown’ period in all the studies which reported medical consultations [28,30,31,32,34,44,45].

**Table 1:** Data extraction table.

Author, Year	Region	Country	Study Aim	Study Design	Data Source	Reported Outcomes	Key Findings
**Adugna et al., (2021) [45]**	Africa	Ethiopia	To determine the effect of COVID-19 on routine HIV care services	Cross-sectional/interrupted time series	District health information system 2 (DHIS-2)	Medical visits	n/a
						ART dispensing	Decline
						Viral load monitoring	n/a
**Bachanas et al. (2022) [41]**	Africa	Botswana, Cameroon, Cote d’Ivoire, Democratic Republic of Congo [DRC], Eswatini, Ethiopia, Kenya, Lesotho, Malawi, Mozambique, Namibia, Nigeria, Rwanda, South Africa, South Sudan, Tanzania, Uganda, Zambia, and Zimbabwe	To evaluate HIV treatment trends prior to and during the COVID-19 pandemic	Cross-sectional study (mixed studies)	Data from PEPFAR-CDC-supported ART sites in 19 African countries	Medical visits	n/a
						ART dispensing	Overall decline with periodic variations within countries
						Viral load monitoring	n/a
**Boyd et al., (2021) [46]**	Africa	Nigeria	To demonstrate the progress of the ART surge in providing and expanding access to HIV services during the COVID-19 pandemic	Cohort study	Nigeria national data repository (NDR)	Medical visit	n/a
						ART dispensing	Overall increase
						Viral load monitoring	n/a
**Celestin et al., (2021) [31]**	Americas	Haiti	To determine the short-term effects of the COVID-19 pandemic on HIV care utilization, service delivery, and continuity of antiretroviral treatment	Cross-sectional study	iSanté EMR system	Medical visits	Decline
						ART dispensing	n/a
						Viral load monitoring	n/a
**Chappell et al., (2023) [38]**	Africa	Zimbabwe	To assess the impact of the COVID-19 pandemic on the provision and uptake of HIV services	Cross-sectional study	District health information	Medical visits	n/a
						ART dispensing	Stable
						Viral load monitoring	n/a
**D’Amato et al., (2020) [33]**	Europe	Italy	To evaluate the impact of the COVID-19 pandemic on HPV vaccination coverage in the general population and in PLWHs	Retrospective cross-sectional study	Coverage data from immunization center	Decline in HPV vaccination	
**Farhat et al., (2022) [43]**	Africa	Burkina Faso, Côte d’Ivoire, and Nigeria	To document the impact of the COVID-19 pandemic on the number of ART initiations and viral load tests conducted before and during the pandemic in adults living with HIV	Cross-sectional study	The international epidemiology database to evaluate AIDS (IeDEA), West Africa	Medical visits	n/a
						ART dispensing	Periodic variations within countries
						Viral load monitoring	Periodic variations within countries
**Harris et al., (2022) [40]**	Africa	Angola, Burundi, Cameroon, Cote d’Ivoire, Democratic Republic of the Congo (DRC), Eswatini, Ethiopia, Kenya, Mozambique, South Sudan, and Zambia	To examine the effect of the COVID-19 pandemic on HIV services using data on the HIV testing and treatment cascade	Cross-sectional study	International Center for AIDS and Treatment Program (ICAP), district health information system (DHIS-2) database	Medical visits	n/a
						ART dispensing	Periodic variations within countries
						Viral load monitoring	n/a
**Htun Nyunt et al., (2021) [35]**	South-east Asia	Myanmar	HIV contingency plan report and its implementation for risk reduction in contracting COVID-19 among PLHIV	Cross-sectional study	Data of national AIDS program	Medical visits	n/a
						ART dispensing	Decline
						Viral load monitoring	Decline
**Lee et al., (2021) [36]**	West Pacific	Australia	To examine whether the COVID-19 pandemic and the lockdown restrictions affected the management of PLHIV	Cross-sectional study	Melbourne Sexual Health Centre (MSHC)	Medical visits	n/a
						ART dispensing	Increase
						Viral load monitoring	n/a
**McGinnis et al., (2021) [28]**	Americas	United States	To compare among PLWH during and prior to the COVID-19 pandemic: (1) HIV healthcare delivery	Cohort study	Veterans aging cohort study (VACS)	Medical visits	Slight decline
						ART dispensing	n/a
						Viral load monitoring	Decline
**Monroe et al., (2022) [29]**	Americas	United States	To assess the impact of COVID-19 on HIV outpatients	Cohort study	DC cohort data	Medical visits	Increase
						ART dispensing	n/a
						Viral load monitoring	n/a
**Norwood et al., (2022) [30]**	Americas	United States	To assess the impact that the COVID-19 pandemic has had on the HIV care continuum	Cross-sectional study	Vanderbilt Comprehensive Care Clinic (VCCC) database, southeastern US	Medical visits	Decline
						ART dispensing	n/a
						Viral load monitoring	n/a
**Osei E et al., (2023) [42]**	Africa	Ghana	Impact of COVID-19 pandemic on tuberculosis and HIV services in Ghana	Cross-sectional study, interrupted time series analysis	District health information management system-2 (DHIMS-2) of Ghana health service	Medical visits	n/a
						ART dispensing	Decline
						Viral load monitoring	n/a
**Pan, et al., (2024) [34]**	Europe	Spain	To estimate the magnitude of the effect of COVID-19 on public healthcare services usage among PLWH	Retrospective cohort study	Public data analysis for health research and innovation program of Catalonia (a quality-controlled information system)	Medical visits	Overall decline
						ART dispensing	n/a
						Viral load monitoring	n/a
**Quiros-Roldan et al., (2020) [32]**	Europe	Italy	To describe the consequences of the pandemic on the HIV continuum of care	Retrospective observational study	Administrative files from Brescia, Lombardy	Medical visit	Decline
						ART dispensing	Decline
						Viral load monitoring	n/a
**Rick et al., (2022) [37]**	Inter-continental study	Africa, Asia, Europe, Latin America, and Caribbean	To describe the impact of the COVID-19 pandemic on HIV testing, the number of appointments, and enrolments for HIV care	Cross-sectional study	Data from the AIDS Healthcare Foundation global quality program sites	Medical visits	Overall decline
						ART dispensing	Overall decline
						Viral load monitoring	n/a
**Schwartz et al., (2020) [44]**	Africa	Uganda	To evaluate the impact of the lockdown on access to medicines	Prospective cohort study	Clinic digital registry	Medical visit	Decline
						ART dispensing	n/a
						Viral load monitoring	n/a
**Thekkur et al., (2021) [39]**	Africa	Zimbabwe	To determine the impact of the COVID-19 pandemic on HIV testing and referral to ART	Cohort study	Routine administrative data	Medical visit	n/a
						ART dispensing	Slight decline
						Viral load monitoring	n/a

n/a = not available—data were not reported in the articles.

Medical consultations of PLHIV differed across several countries in our review, with marked inter-country variations of in-person visitations, with a decline in Latin America and Africa, and a slight increase in medical visits in Asia and Europe [37]. Virtual consultations increased during the pandemic, with a remarkably greater proportion of PLHIV receiving care remotely in 2020 compared to 2019. The transition to virtual visits coincided with the declaration of the restriction of movement in March 2020. A cross-sectional study from the United States [30] which evaluated medical visits based on quarterly visits from 2017 to 2020 described that there was a profound decrease (23.5%) in medical visits of newly diagnosed HIV clients in the second quarter of 2020 (with the declaration of lockdown) compared to previous years. Similarly, a retrospective study of a large cohort of HIV clients in Spain [34] discovered an average medical visits per day of 357 during the lockdown in 2020 compared to 449 daily visits before the lockdown period. They also observed that the average number of visits to primary healthcare centers was at its highest during the pandemic in 2020 when compared to visits to other levels of service delivery units such as HIV units and emergency departments.

  - Women living with HIV

Women living with HIV were less likely to receive in-person consultations compared to men. Women received more virtual consultations during the pandemic than the male counterparts [28]. Also, women significantly missed their scheduled routine medical appointments compared to men during this period [32], as seen in Table 2 below.

2. **Anti-retroviral therapy (ART) dispensing**
  - General population living with HIV

Overall, ART dispensing markedly decreased across most studies in different regions during the lockdown, as seen in Table 1. ART dispensing and initiation services decreased exponentially by more than three times with the declaration of the restriction measures (lockdown) in 2020 compared to the same period in 2019 [45]. Most of the studies identified an initial decrease in ART dispensing at the beginning of the restriction of movement, as seen in Table 1. An inter-continental study [37] observed an overall decline in enrollments for ART, with a 38.2% decrease in fresh enrollments in 2020. Enrollments in HIV programs serve as a medium for ART dispensing services which involve initiation and refill services. Looking closely at the continents shows a decrease in enrollments in Africa, Asia, and Latin America during the pandemic; however, an increase in European countries occurred. Two studies in Nigeria and Myanmar [35,46] described a sudden rise in ART initiation particularly in June 2020; however, there was an increase in ART uptake in Nigeria from February to September 2020, with a negligible decline during the strict restriction of movement from March to May 2020 [46]. In Australia, the number of ART medications dispensed decreased in 2020 compared to 2019; however, PLHIV had an abruptly increased uptake of ART medications in 2020 during the period of the declaration of the lockdown through an indirect approach via postage delivery compared to the previous year [36]. Postage delivery of ART remained relatively stable before and during the pandemic, with an abrupt increase.

Although there was an increasing trend of new initiations in four countries (Cameroun, Mozambique, Nigeria, and Tanzania) in a study of the impact of COVID-19 on PLHIV in 19 African countries, an overall decline in new ART initiation was observed during the pandemic in 2020 which was marked by a sharp decline during the lockdown period from April to June of the same year [41]. Another study across 11 sub-Saharan African countries revealed that that there was an increase in ART initiation just before the lockdown in March 2020 compared to previous quarters. Similarly, the proportion of PLHIV who received more than three months’ multi-month dispensing increased progressively over the one-year period from October 2019 to September 2020 [40]. This is also similar to a cohort study in Nigeria where the multi-month dispensing uptake increased from 60% in April to 98% in August 2020 [46].

  - Women living with HIV

In a study in West African clinics, Ivorian women were less likely to initiate ART medications [43]. Although there was a statistically significant decline in the number of people currently on ART among the general population, the number of Zimbabwean WLHIV on ART remained stable during the pandemic [38].

3. **Viral load monitoring**
  - General population living with HIV

Three studies, one in United States [28], one in south-east Asia [35], and one in sub-Saharan Africa [43], reported a decline in viral load testing of PLHIV. According to McGinnis, K. A., et al. [28], using a national cohort of PLHIV, there was a drop in viral load tests from 82% in 2019 to 74% in 2020. In south-east Asia, Myanmar, PLHIV could not monitor their viral load routinely due to a delay in the supply of test tools necessary for viral load monitoring due to the restriction of movement which affected the transportation system [35]. In sub-Saharan African countries, the level of strict measures implemented in the restriction of movement and monitoring of viral load in PLHIV showed a significant overall decline during the pandemic in countries such as Burkina Faso, Ivory Coast, and Nigeria; however, the overall trend of the proportion of PLHIV who received viral load tests increased during all the four quarters of the one-year study period (October 2019 to September 2020) across the 11 sub-Saharan African countries [40].

Other services such as HPV vaccination [33] reported that the number of doses of HPV vaccination administered during the pandemic was lower compared to the pre-pandemic era. Although marginally significant, there were lower doses of HPV vaccine administered between January and May 2020 compared to the same period in 2019.

  - Women living with HIV

Although there was an overall decline in the testing services for PLHIV, McGinnis et al., in their study in the United States, described that WLHIV were less likely to have their viral load routinely tested when compared to men [28]. Similarly, in Zimbabwe, there was a drop in the HIV testing of pregnant women in April 2020 during the pandemic [38].

## 4. Discussion

The public health measures introduced during the COVID-19 pandemic, such as the unusual restriction of movement to prevent the spread and mitigate the undesirable health consequences of SARS-CoV-2 [47], came with the challenge of limited access to care services with attendance alterations based on the amount of healthcare use. Our study reveals that, during the COVID-19 pandemic, there was a substantial decrease in the use of healthcare services by PLHIV, with ART dispensing showing a marked decline followed by viral load monitoring. Most of the countries under review experienced a decrease in care services; however, certain HIV care services were functional during the pandemic. Our study shows that prevention of mother-to-child transmission (PMTCT) services remained stable during the pandemic, suggesting that care services embedded within the healthcare systems will invariably remain consistent amidst uncertainties and health emergencies such as pandemics. This is corroborated by previous research, according to which deviation from vertical preventive health interventions toward integrating and systematizing HIV/AIDS care programs within the streams of the healthcare system presents an effective method for the sustainability of HIV prevention programs [48]. Thus, facilitating the identification and prevention of possible incident cases of HIV is critical for the attainment of the sustainable development goal target of HIV eradication—“The Project 2030 goal” [49].

Previous health reports have suggested that the proportion of PLHIV who were receiving ART monthly remained relatively constant from April to September 2020 [50]; however, our study discovered that PLHIV faced a decline in ART services due to the disruptions caused by the pandemic in different regions of the world. The reduction in the uptake of ART services could be attributable to the skipped medical consultations in most countries, especially in the sub-Saharan African region, where ART initiations often occur after the medical consultations [45]. The case is different in other regions like Australia, where the delivery of ART could be achieved via mail services [36], indicative of the need to re-invent strategies that are efficient for the administration of HIV services to key populations in times of health emergencies, particularly in the sub-Saharan African region where the burden of HIV persists the most [51]. PLHIV have often been shown to experience a decrease in healthcare services when compared to people of the same demographics who are without HIV [52]. In our study, the absence of adequate supply chain networks of medical services during the pandemic was one of the reasons for the decline in health service uptake (ART dispensing and viral load monitoring) among PLHIV. The reduction in health utilization could present some levels of stressful life conditions to this population, as the fear of an increased viral load due to the lack of monitoring accompanied by the apprehension of being infected with the SARS-CoV-2 virus could affect their overall health and heighten comorbidities among PLHIV [53,54], corroborated by previous studies [13]. This buttresses the view that these disruptions in HIV preventive services during the pandemic periods could lead to a higher incidence of HIV cases when compared to the pre-pandemic period [55], an area for further research.

Furthermore, the majority of the studies in our review were from the sub-Saharan African (SSA) region, with data collection through programmatically implemented HIV investment projects. The data from these projects are currently one of the most viable sources of HIV data in the region. Thus, it is necessary to encourage data ownership with the support of the indigenous HIV data collection machinery within the health system to enable its reliability. Our study shows that most African countries had an initial sudden decline in ART initiation services at the declaration of the lockdown, accompanied by an upsurge in ART initiation. Also, the launch of the strategic action plan and implementation of the multi-month dispensing of ART (a form of differentiated service delivery) during the early pandemic era led to a minimal shortage of ART with attendance improvement in HIV viral load monitoring. The multi-month dispensing of ART, unlike the traditional routine monthly HIV appointments [56], was instrumental to the increase of ART dispensing in some African regions during the pandemic. Most of these HIV care actions could be traceable to the commitment and concerted efforts of the government and collaborations with international support programs and donors toward the reduction of the burden of HIV/AIDS in the region [57].

Our study also examined the impact of COVID-19 on PLHIV with a convergent view on women of childbearing age (15–45 years) and above, as they represent a crucial demographic [58]. We discovered that there is a lack of research studies regarding women populations living with HIV/AIDS. This limited research on the women populations living with HIV/AIDS could be a pointer to inadequate health system structures for women’s health, especially during emergency health situations. Our review shows that women living with HIV rarely continued medical visits, seldom had their viral load tested, and were less likely to initiate ART during the pandemic, actions attributed to the heightened stigma and discrimination among women populations and to the widening gender inequalities. This represents a major barrier that PLHIV had to contend with during the COVID-19 pandemic, thereby increasing their vulnerability during emergency situations [59,60] and leading to the uptake of the prevailing challenges of HIV care by women. These public health issues regarding the state healthcare preparedness for vulnerable populations serve as a clarion call to health organizations and alliances to better design innovative strategies in collaboration with community-based organizations, women groups, civil societies, and government agencies tailored toward ending discrimination against women. Community-based partnerships and the use of culturally adapted social support programs through faith-based organizations that encourage women to receive healthcare services and those that strengthen women’s agency could be beneficial in addressing gender disparities [61].

Our findings show that primary care played an essential role during the pandemic. Although there was a general decline in medical consultations at hospitals and emergency departments during the pandemic, HIV patients received more care at primary healthcare centers [34]. This could suggest that increasing access to primary healthcare during pandemics, if well implemented, may stimulate and encourage PLHIV to seek and receive care. This highlights the role of the primary healthcare centers (PHCs) as the bedrock of health systems, and the most guaranteed approach to ensure universal health coverage [62,63]. Therefore, there is a need to continue promoting the strengthening of PHC systems, especially in developing countries. Our study also identified that medical consultations during the COVID-19 pandemic were more frequently carried out remotely, as virtual consultations were at their highest [29]. Although there was a decline in face-to-face medical visits in our review, remote medical consultations increased during the pandemic, as reported in a previous study [64]. Remote consultations are gradually becoming widely recommended during pandemics, as they offer greater patient satisfaction and are equally effective compared to in-person visitation [65]. Thus, the paradigm shift from traditional face-to-face to virtual consultations has become imperative, particularly during pandemics [66]. Therefore, there is a need to continually maximize the potential of telemedicine by harnessing current remote platforms and improving those already existing to ensure efficient user experience for guaranteed quality service delivery during pandemics.

### Strengths and Limitations

The strength of the study is that, to the best of the reviewers’ knowledge, this is one of the first systematic reviews examining the impact of the COVID-19 pandemic on PLHIV with a focus on women living with HIV/AIDS. Also, the comprehensive search strategy of the selected database ensured that all studies were included in the review. Furthermore, the description of the decline in healthcare utilization could be generalizable, as it was specific to the period (lockdown period), although the exact dates could have varied from country to country.

There are several limitations in our study. Certain prevailing context-specific conditions could have been at play during the advent of the pandemic. Thus, the overall decline in the health service utilization among PLHIV in our study should be interpreted with caution because the reduction in care utilization may not only be attributable to the consequences of the COVID-19 pandemic. Therefore, the study does not describe causality. Also, the heterogeneity of the studies, in terms of designs and variables considered as outcomes, included in the review prevented the conduction of a meta-analysis, thus leading to the narrative synthesis of the study. There could be a potential risk of bias in our study, as only studies in the English language and quantitative studies were included in the study.

## 5. Conclusions

Our study describes the impact of the COVID-19 pandemic on PLHIV, as well as carrying out a sub-group analysis, narrowing it down to women living with HIV/AIDS. Although we observed variations in healthcare utilization at different periods during the COVID-19 pandemic in some countries, there was an overall decline in healthcare utilization among PLHIV. Furthermore, there are scarce studies and research regarding women living with HIV/AIDS worldwide during the COVID-19 pandemic, indicating the need to prioritize women’s health during health emergencies through intensified public health preparedness. A major finding of this work is that primary care played a remarkable role during the pandemic, reinforcing the need to support strategies toward strengthening systems and revitalizing primary care centers to deliver efficient healthcare services for PLHIV and vulnerable populations.

## Figures and Tables

**Table 2:** Sub-group analysis of papers including specific data on women.

Author, Year	Country	OUTCOME		
		Medical Visit	ART Dispensing	Viral Load Monitoring
**Chappell E., et al. (2023) [38]**	Zimbabwe	n/a	Stable	Reduction in VL monitoring
**Farhat et al. (2020) [43]**	Burkina Faso, Ivory Coast, and Nigeria	n/a	Unlikely to initiate treatment	n/a
**Quiros-Roldan et al. (2020) [32]**	Italy	Fewer visits	n/a	n/a
**McGinnis et al. (2021) [28]**	11 sub-Saharan African countries	Fewer visits	n/a	Unlikely to be tested

n/a = not available—data were not reported in the articles.

## Data Availability

No new data were created or analyzed in this study. Data sharing is not applicable to this article. The original contributions presented in the study are included in the article. Further inquiries can be directed to the corresponding author.

## Supplementary Materials

The following supporting information can be downloaded at: https://www.mdpi.com/article/10.3390/medicina61010111/s1, Table S1: Search strategy; Table S2: Quality assessment.

## References

[1] Spiteri G., Fielding J., Diercke M., Campese C., Enouf V., Gaymard A., Bella A., Sognamiglio P., Sierra Moros M.J., Riutort A.N. (2020). First cases of coronavirus disease 2019 (COVID-19) in the WHO European Region, 24 January to 21 February 2020. Eurosurveillance.

[2] World Health Organization (2024). WHO Coronavirus (COVID-19) Dashboard. https://covid19.who.int/.

[3] Cucinotta D., Vanelli M. (2020). WHO Declares COVID-19 a Pandemic. Acta Biomed..

[4] WHO (World Health Organization) (2020). Overview of Public Health and Social Measures in the Context of COVID-19: Interim Guidance, 18 May 2020.

[5] Nicola M., Alsafi Z., Sohrabi C., Kerwan A., Al-Jabir A., Iosifidis C., Agha M., Agha R. (2020). The socio-economic implications of the coronavirus pandemic (COVID-19): A review. Int. J. Surg..

[6] Moynihan R., Sanders S., Michaleff Z.A., Scott A.M., Clark J., To E.J., Jones M., Kitchener E., Fox M., Johansson M. (2021). Impact of COVID-19 pandemic on utilisation of healthcare services: A systematic review. BMJ Open.

[7] William Lodge I.I. (2020). COVID-19, HIV, and migrant workers: The double burden of the two viruses. AIDS Patient Care STDs.

[8] Ballester-Arnal R., Gil-Llario M.D. (2020). The virus that changed Spain: Impact of COVID-19 on people with HIV. AIDS Behav..

[9] SeyedAlinaghi S., Mirzapour P., Pashaei Z., Afzalian A., Tantuoyir M.M., Salmani R., Maroufi S.F., Paranjkhoo P., Maroufi S.P., Badri H. (2023). The impacts of COVID-19 pandemic on service delivery and treatment outcomes in people living with HIV: A systematic review. AIDS Res. Ther..

[10] Mude W., Mwenyango H., Preston R., O’Mullan C., Vaughan G., Jones G. (2024). HIV testing disruptions and service adaptations during the COVID-19 pandemic: A systematic literature review. AIDS Behav..

[11] Erly S., Menza T.W., Granillo L., Navejas M., Udeagu C.-C.N., Brady K., Hixson L.K., Raj-Sing S., Nassau T., Kaasa C. (2022). Impact of COVID-19 on People Living with HIV: Data from Five Medical Monitoring Project Sites, 2020–2022. JAIDS J. Acquir. Immune Defic. Syndr..

[12] Weerasuria M., Ko C., Ehm A., O’Bryan J., McMahon J., Woolley I., Hoy J., Lau J. (2021). The impact of the COVID-19 pandemic on people living with HIV in Victoria, Australia. AIDS Res. Hum. Retroviruses.

[13] Lee K.W., Ang C.S., Lim S.H., Siau C.S., Ong L.T.D., Ching S.M., Ooi P.B. (2022). Prevalence of mental health conditions among people living with HIV during the COVID-19 pandemic: A rapid systematic review and meta-analysis. HIV Med..

[14] Dusina A., Lombardi F., Tamburrini E., Onorati F., Petrucci M., Di Giambenedetto S. (2023). Home Care Assistance: Has COVID-19 had an Impact on the Complex Management of HIV Patients?. AIDS Behav..

[15] Pifarré i Arolas H., Acosta E., López-Casasnovas G., Lo A., Nicodemo C., Riffe T., Myrskylä M. (2021). Years of life lost to COVID-19 in 81 countries. Sci. Rep..

[16] Girum T., Wasie A., Lentiro K., Muktar E., Shumbej T., Difer M., Shegaze M., Worku A. (2018). Gender disparity in epidemiological trend of HIV/AIDS infection and treatment in Ethiopia. Arch. Public Health.

[17] Wu C., Qian Y. (2022). The gender peak effect: Women are most vulnerable to infections during COVID-19 peaks. Front. Public Health.

[18] Kuandyk A., Ortega M.-A., Ntegwa M.J., Sarria-Santamera A. (2024). Impact of the COVID-19 pandemic on access to and delivery of maternal and child healthcare services in low-and middle-income countries: A systematic review of the literature. Front. Public Health.

[19] Penna A.L., de Aquino C.M., Pinheiro M.S.N., Do Nascimento R.L.F., Farias-Antúnez S., Araújo D.A.B.S., Mita C., Machado M.M.T., Castro M.C. (2023). Impact of the COVID-19 pandemic on maternal mental health, early childhood development, and parental practices: A global scoping review. BMC Public Health.

[20] Otu A., Yaya S. (2022). Uncovering the collateral impacts of COVID-19 on maternal mental health. Reprod. Health.

[21] Ghanotakis E., Peacock D., Wilcher R. (2012). The importance of addressing gender inequality in efforts to end vertical transmission of HIV. J. Int. AIDS Soc..

[22] Page M.J., McKenzie J.E., Bossuyt P.M., Boutron I., Hoffmann T.C., Mulrow C.D., Shamseer L., Tezlaff J.M., Akl E.A., Brennan S.E. (2021). The PRISMA 2020 statement: An updated guideline for reporting systematic reviews. BMJ.

[23] (2023). National Heart, Lung, and Blood Study Quality Assessment Tools|National Heart, Lung, and Blood Institute (NHLBI). https://nhlbi.nih.gov/health-topics/study-quality-assessment-tools.

[24] Coughlan M., Cronin P., Ryan F. (2007). Step-by-step guide to critiquing research. Part 1: Quantitative research. Br. J. Nurs..

[25] Popay J. (2006). Guidance on the Conduct of Narrative Synthesis in Systematic Reviews. ESRC Methods Programme.

[26] Mugo C., Adedokun O., Alo O.D., Ezeokafor N., Adeyemi S., Kpamor Z., Madueke L., James E., Adebajo S.B., Semo B.-w. (2024). Effects of the COVID-19 pandemic on HIV service delivery and viral suppression: Findings from the SHARP program in Northern Nigeria. PLoS ONE.

[27] Zhu W., Huang Y.-l.A., Wiener J., Neblett-Fanfair R., Kourtis A.P., Hall H.I., Hoover K.W. (2022). Impact of the coronavirus disease 2019 pandemic on prescriptions for antiretroviral drugs for HIV treatment in the United States, 2019–2021. AIDS.

[28] McGinnis K.A., Skanderson M., Justice A.C., Akgün K.M., Tate J.P., King J.T., Rentsch C.T., Marconi V.C., Hsieh E., Ruser C. (2021). HIV care using differentiated service delivery during the COVID-19 pandemic: A nationwide cohort study in the US Department of Veterans Affairs. J. Int. AIDS Soc..

[29] Monroe A.K., Xiao J., Greenberg A.E., Levy M.E., Temprosa M., Resnik J.B., Castel A.D. (2022). Risk of severe COVID-19 Disease and the pandemic’s impact on service utilization among a longitudinal cohort of persons with HIV-Washington, DC. AIDS Behav..

[30] Norwood J., Kheshti A., Shepherd B.E., Rebeiro P.F., Ahonkhai A., Kelly S., Wanjalla C. (2022). The impact of COVID-19 on the HIV care continuum in a large urban southern clinic. AIDS Behav..

[31] Celestin K., Allorant A., Virgin M., Marinho E., Francois K., Honoré J.G., White C., Valles J.S., Perrin G., De Kerorguen N. (2021). Short-term effects of the COVID-19 pandemic on HIV care utilization, service delivery, and continuity of HIV antiretroviral treatment (ART) in Haiti. AIDS Behav..

[32] Quiros-Roldan E., Magro P., Carriero C., Chiesa A., El Hamad I., Tratta E., Fazio R., Formenti B., Castelli F. (2020). Consequences of the COVID-19 pandemic on the continuum of care in a cohort of people living with HIV followed in a single center of Northern Italy. AIDS Res. Ther..

[33] D’Amato S., Nunnari G., Trimarchi G., Squeri A., Cancellieri A., Squeri R., Pellicanò G.F. (2022). Impact of the COVID-19 pandemic on HPV vaccination coverage in the general population and in PLWHs. Eur. Rev. Med. Pharmacol. Sci..

[34] Pan Y.-H., Nomah D.K., Montoro-Fernandez M., Moreno-Fornés S., Díaz Y., Aceitón J., Bruguera A., Llibre J.M., Domingo P., Imaz A. (2024). The impact of the COVID-19 pandemic on healthcare services utilization among people living with HIV in Catalonia, Spain: A population-based cohort study. Enfermedades Infecc. Y Microbiol. Clin. (Engl. Ed.).

[35] Htun Nyunt O., Wan N.M.A., Soan P., Tawil O., Lwin M.K., Hsan M.T.A., Win K.M., Mesquita F. (2021). How Myanmar is working to maintain essential services for people living with HIV and key populations during the COVID-19 pandemic. J. Int. Assoc. Provid. AIDS Care (JIAPAC).

[36] Lee D., Chow E.P.F., Aguirre I., Fairley C.K., Ong J.J. (2021). Access to HIV antiretroviral therapy among people living with HIV in Melbourne during the COVID-19 pandemic. Int. J. Environ. Res. Public Health.

[37] Rick F., Odoke W., Van Den Hombergh J., Benzaken A.S., Avelino-Silva V.I. (2022). Impact of coronavirus disease (COVID-19) on HIV testing and care provision across four continents. HIV Med..

[38] Chappell E., Chimwaza A., Manika N., Wedderburn C.J., Mupambireyi Nenguke Z., Gannon H., Cowan F., Gibb T., Heys M., Fitzgerald F. (2023). Impact of the COVID-19 pandemic on the provision and uptake of services for the prevention of mother-to-child transmission of HIV in Zimbabwe. PLOS Glob. Public Health.

[39] Thekkur P., Takarinda K.C., Timire C., Sandy C., Apollo T., Kumar A.M.V., Satyanarayana S., Shewade H.D., Khogali M., Zachariah R. (2021). Operational research to assess the real-time impact of COVID-19 on TB and HIV services: The experience and response from health facilities in Harare, Zimbabwe. Trop. Med. Infect. Dis..

[40] Harris T.G., Jaszi E., Lamb M.R., Laudari C.A., Furtado M.L.M., Nijirazana B., Aimé N., Loni Ekali G., Ebiama Lifanda L., Brou H. (2022). Effects of the coronavirus disease 2019 pandemic on human immunodeficiency virus services: Findings from 11 sub-Saharan African countries. Clin. Infect. Dis..

[41] Bachanas P.J., Chun H.M., Mehta N., Aberle-Grasse J., Parris K., Sherlock M.W., Lloyd S., Zeh C., Makwepa D.K., Kapanda M.L. (2022). Protecting the gains: Analysis of HIV treatment and service delivery programme data and interventions implemented in 19 African countries during COVID-19. J. Int. AIDS Soc..

[42] Osei E., Amu H., Kye-Duodu G., Kwabla M.P., Danso E., Binka F.N., Kim S.Y. (2023). Impact of COVID-19 pandemic on Tuberculosis and HIV services in Ghana: An interrupted time series analysis. PLoS ONE.

[43] Farhat J.B., Tiendrebeogomd T., Malateste K., Poda A., Minga A., Messou E., Chenal H., Ezechi O., Ofotokun I., k Ekouevi D. (2022). Effects of the COVID-19 pandemic on ART initiation and access to HIV viral load monitoring in adults living with HIV in West Africa: A regression discontinuity analysis. JAIDS J. Acquir. Immune Defic. Syndr..

[44] Schwartz J.I., Muddu M., Kimera I., Mbuliro M., Ssennyonjo R., Ssinabulya I., Semitala F.C. (2021). Impact of a COVID-19 national lockdown on integrated care for hypertension and HIV. Glob Heart..

[45] Adugna A., Azanaw J., Sharew Melaku M. (2021). The effect of COVID-19 on routine HIV care services from health facilities in Northwest Ethiopia. HIV/AIDS-Res. Palliat. Care.

[46] Boyd A.T., Jahun I., Dirlikov E., Greby S., Odafe S., Abdulkadir A., Odeyemi O., Dalhatu I., Ogbanufe O., Abutu A. (2021). Expanding access to HIV services during the COVID-19 pandemic—Nigeria, 2020. AIDS Res. Ther..

[47] WHO (World Health Organization) (2021). Infection Prevention and Control During Health Care When Coronavirus Disease (COVID-19) Is Suspected or Confirmed: Interim Guidance, 12 July 2021.

[48] Assefa Y., Tesfaye D., Van Damme W., Hill P.S. (2018). Effectiveness and sustainability of a diagonal investment approach to strengthen the primary health-care system in Ethiopia. Lancet.

[49] Assefa Y., Gilks C.F. (2020). Ending the epidemic of HIV/AIDS by 2030: Will there be an endgame to HIV, or an endemic HIV requiring an integrated health systems response in many countries?. Int. J. Infect. Dis..

[50] UNAIDS Prevailing Against Pandemics by Putting People at the Center—World AIDS Day Report 2020. https://www.unaids.org/en/resources/documents/2020/prevailing-against-pandemics.

[51] James S.L., Abate D., Abate K.H., Abay S.M., Abbafati C., Abbasi N., Abbastabar H., Abd-Allah F., Abdela J., Abdelalim A. (2018). Global, regional, and national incidence, prevalence, and years lived with disability for 354 diseases and injuries for 195 countries and territories, 1990–2017: A systematic analysis for the Global Burden of Disease Study 2017. Lancet.

[52] Cooley S.A., Nelson B., Doyle J., Rosenow A., Ances B.M. (2021). Collateral damage: Impact of SARS-CoV-2 pandemic in people living with HIV. J. NeuroVirol..

[53] Vizcarra P., Pérez-Elías M.J., Quereda C., Moreno A., Vivancos M.J., Dronda F., Casado J.L., Moreno S., Pérez-Elías M.J., Fortún J. (2020). Description of COVID-19 in HIV-infected individuals: A single-centre, prospective cohort. Lancet HIV.

[54] Suwanwongse K., Shabarek N. (2020). Clinical features and outcome of HIV/SARS-CoV-2 coinfected patients in The Bronx, New York city. J. Med. Virol..

[55] Viguerie A., Jacobson E.U., Hicks K.A., Bates L., Carrico J., Honeycutt A., Lyles C., Farnham P.G. (2024). Assessing the Impact of COVID-19 on HIV Outcomes in the United States: A Modeling Study. Sex. Transm. Dis..

[56] Preko P., Shongwe S., Abebe A., Vandy A., Aly D., Boraud F. (2020). Rapid adaptation of HIV differentiated service delivery program design in response to COVID-19: Results from 14 countries in sub-Saharan Africa. AIDS.

[57] Olakunde B.O., Adeyinka D.A. (2017). Test-and-treat approach to ending HIV epidemic in Nigeria: Current status and future prospects of domestic funding. HIV AIDS Review. Int. J. HIV-Relat. Probl..

[58] Verma S., Carter E.B., Mysorekar I.U. (2020). SARS-CoV2 and pregnancy: An invisible enemy?. Am. J. Reprod. Immunol..

[59] Krier S., Bozich C., Pompa R., Friedman M.R. (2020). Assessing HIV-Related Stigma in Healthcare Settings in the Era of the COVID-19 Pandemic, Pittsburgh, Pennsylvania. AIDS Behav..

[60] Siriwardhane P., Khan T. (2021). The gendered nature of the risk factors of the COVID-19 pandemic and gender equality: A literature review from a vulnerability perspective. Sustainability.

[61] Belgrave F.Z., Abrams J.A. (2016). Reducing disparities and achieving equity in African American women’s health. Am. Psychol..

[62] Wenham C., Katz R., Birungi C., Boden L., Eccleston-Turner M., Gostin L. (2019). Global health security and universal health coverage: From a marriage of convenience to a strategic, effective partnership. BMJ Glob. Health.

[63] De Maeseneer J., Li D., Palsdottir B., Mash B., Aarendonk D., Stavdal A., Moosa S., Decat P., Kiguli-Malwadde E., Ooms G. (2020). Universal health coverage and primary health care: The 30 by 2030 campaign. Bull. World Health Organ..

[64] Xu S., Glenn S., Sy L., Qian L., Hong V., Ryan D.S., Jacobsen S. (2021). Impact of the COVID-19 Pandemic on Health Care Utilization in a Large Integrated Health Care System: Retrospective Cohort Study. J. Med. Internet Res..

[65] Mariani M.V., Pierucci N., Forleo G.B., Schiavone M., Bernardini A., Gasperetti A., Mitacchione G., Mei M., Giunta G., Piro A. (2023). The Feasibility, Effectiveness and Acceptance of Virtual Visits as Compared to In-Person Visits among Clinical Electrophysiology Patients during the COVID-19 Pandemic. J. Clin. Med..

[66] Haleem A., Javaid M., Singh R.P., Suman R. (2021). Telemedicine for healthcare: Capabilities, features, barriers, and applications. Sens. Int..

