# Supplementary material for: Impact of COVID-19 on Utilization of Healthcare Services Among People Living with HIV (PLHIV): A Systematic Review
Source: Medicina (Kaunas). 2025 Jan 14;61(1):111. doi: 10.3390/medicina61010111 (PMC11766806; doi:10.3390/medicina61010111)
Supplement: Supplementary file 1 [file medicina-61-00111-s001.zip › Quality Assessment.pdf]

Supplementary Table

Table S2: Quality Assessment

S/N	Criteria	1. Was the research question or objective in this paper clearly stated?	2. Was the study population clearly specified and defined?	3. Was the participation rate of eligible persons at least 50%?	4. Were all the subjects selected or recruited from the same or similar populations (including the same time period)? Were inclusion and exclusion criteria for being in the study pre specified and applied uniformly to all participants?	5. Was a sample size justification, power description, or variance and effect estimates provided?	6. For the analyses in this paper, were the exposure(s) of interest measured prior to the outcome(s) being measured?
1	Adugna et al., (2021) **[45]**	YES	YES	CD	YES	NA	CD
2	Bachanas et al., (2022) **[41]**	YES	YES	CD	YES	NA	CD
3	Boyd et al., (2021) **[46]**	YES	YES	CD	YES	NA	CD
4	Celestin et al., (2021) **[31]**	YES	YES	CD	YES	NA	CD
5	Chappell et al., (2023) **[38]**	YES	YES	CD	YES	NA	CD
6	D'Amato et al., (2022) **[33]**	YES	YES	CD	YES	NA	CD
7	Farhat et al., (2022) **[43]**	YES	YES	CD	YES	NA	CD
8	Harris et al., (2022) **[40]**	YES	YES	CD	YES	NA	CD
9	Htun Nyunt et al., (2021) **[35]**	YES	YES	CD	YES	NA	CD
10	Lee et al., (2021) **[36]**	YES	YES	CD	YES	NA	CD
11	McGinnis et al., (2021) **[28]**	YES	YES	CD	YES	NA	CD
12	Monroe et al., (2022) **[29]**	YES	YES	CD	YES	NA	CD
13	Norwood et al., 2022) **[30]**	YES	YES	CD	YES	NA	CD
14	Osei et al., (2023) **[42]**	YES	YES	CD	YES	NA	CD
15	Pan et al., (2024) **[34]**	YES	YES	CD	YES	NA	CD
16	Quiros-Roldan et al., (2020) **[32]**	YES	YES	CD	YES	NA	CD
17	Rick et al., (2022) **[37]**	YES	YES	CD	YES	NA	CD
18	Schwartz et al., (2021) **[44]**	YES	YES	CD	YES	NA	CD
19	Thekkur et al., (2021) **[39]**	YES	YES	CD	YES	NA	CD

Table S2: Quality Assessment (cont'd)

S/N	Criteria	7. Was the timeframe sufficient so that one could reasonably expect to see an association between exposure and outcome if it existed?	8. For exposures that can vary in amount or level, did the study examine different levels of the exposure as related to the outcome (e.g., categories of exposure, or exposure measured as continuous variable)?	9. Were the exposure measures (independent variables) clearly defined, valid, reliable, and implemented consistently across all study participants?	10. Was the exposure(s) assessed more than once over time?
1	Adugna et al., (2021) **[45]**	YES	NA	YES	YES
2	Bachanas et al., (2022) **[41]**	YES	NA	YES	YES
3	Boyd et al., (2021) **[46]**	YES	NA	YES	YES
4	Celestin et al., (2021) **[31]**	YES	NA	YES	YES
5	Chappell et al., (2023) **[38]**	YES	NA	YES	YES
6	D'Amato et al., (2022) **[33]**	YES	NA	YES	YES
7	Farhat et al., (2022) **[43]**	YES	NA	YES	YES
8	Harris et al., (2022) **[40]**	YES	NA	YES	YES
9	Htun Nyunt et al., (2021) **[35]**	YES	NA	YES	YES
10	Lee et al., (2021) **[36]**	YES	NA	YES	YES
11	McGinnis et al., (2021) **[28]**	YES	NA	YES	YES
12	Monroe et al., (2022) **[29]**	YES	NA	YES	YES
14	Norwood et al., 2022) **[30]**	YES	NA	YES	NA
14	Osei et al., (2023) **[42]**	YES	NA	YES	YES
15	Pan et al., (2024) **[34]**	YES	NA	YES	YES
16	Quiros-Roldan et al., (2020) **[32]**	YES	NA	YES	YES
17	Rick et al., (2022) **[37]**	YES	NA	YES	YES
18	Schwartz et al., (2021) **[44]**	YES	NA	YES	YES
19	Thekkur et al., (2021) **[39]**	YES	NA	YES	YES

Table S2: Quality Assessment (cont'd)

S/N	Criteria	11. Were the outcome measures (dependent variables) clearly defined, valid, reliable, and implemented consistently across all study participants?	12. Were the outcome assessors blinded to the exposure status of participants?	13. Was loss to follow-up after baseline 20% or less?	14. Were key potential confounding variables measured and adjusted statistically for their impact on the relationship between exposure(s) and outcome(s)?
1	Adugna et al., (2021) **[45]**	YES	NA	NA	CD
2	Bachanas et al., (2022) **[41]**	YES	NA	NA	CD
3	Boyd et al., (2021) **[46]**	YES	NA	NA	CD
4	Celestin et al., (2021) **[31]**	YES	NA	NA	CD
5	Chappell et al., (2023) **[38]**	YES	NA	NA	CD
6	D'Amato et al., (2022) **[33]**	YES	NA	NA	CD
7	Farhat et al., (2022) **[43]**	YES	NA	NA	CD
8	Harris et al., (2022) **[40]**	YES	NA	NA	CD
9	Htun Nyunt et al., (2021) **[35]**	YES	NA	NA	CD
10	Lee et al., (2021) **[36]**	YES	NA	NA	CD
11	McGinnis et al., (2021) **[28]**	YES	NA	NA	CD
12	Monroe et al., (2022) **[29]**	YES	NA	NA	CD
13	Norwood et al., 2022) **[30]**	YES	NA	NA	CD
14	Osei et al., (2023) **[42]**	YES	NA	NA	CD
15	Pan et al., (2024) **[34]**	YES	NA	NA	CD
16	Quiros-Roldan et al., (2020) **[32]**	YES	NA	NA	CD
17	Rick et al., (2022) **[37]**	YES	NA	NA	CD
18	Schwartz et al., (2021) **[44]**	YES	NA	NA	CD
19	Thekkur et al., (2021) **[39]**	YES	NA	NA	CD
