# Supplementary material for: Impact of COVID-19 on Utilization of Healthcare Services Among People Living with HIV (PLHIV): A Systematic Review
Source: Medicina (Kaunas). 2025 Jan 14;61(1):111. doi: 10.3390/medicina61010111 (PMC11766806; doi:10.3390/medicina61010111)
Supplement: Supplementary file 1 [file medicina-61-00111-s001.zip › Supplementary Tables.pdf]

Supplementary Table

Table S1: Search Strategy

**Search Headings**	
Covid-19; COVID; coronavirus; corona	
AND	
Healthcare services; health care services; Health services; medical care; medical services	
AND	
1)sexually transmitted disease; STD	(TITLE-ABS-KEY (covid-19) OR TITLE-ABS-KEY (covid) OR TITLE-ABS-KEY (coronavirus) OR TITLE-ABS-KEY (corona)) AND (TITLE-ABS-KEY (sexually AND transmitted AND disease) OR TITLE-ABS-KEY (std)) AND (TITLE-ABS-KEY (healthcare AND services) OR TITLE-ABS-KEY (health AND care AND services) OR TITLE-ABS-KEY (health AND services) OR TITLE-ABS-KEY (medical AND care) OR TITLE-ABS-KEY (medical AND services)) AND PUBYEAR > 2019 AND PUBYEAR < 2025 AND (LIMIT-TO (DOCTYPE , "ar")) AND (LIMIT-TO (LANGUAGE , "English"))
2) human immunodeficiency virus; HIV; HIV-1; HIV-2	(TITLE-ABS-KEY (covid-19) OR TITLE-ABS-KEY (covid) OR TITLE-ABS-KEY (coronavirus) OR TITLE-ABS-KEY (corona)) AND (TITLE-ABS-KEY (human AND immunodeficiency AND virus) OR TITLE-ABS-KEY (hiv) OR TITLE-ABS-KEY (hiv-1) OR TITLE-ABS-KEY (hiv-2)) AND (TITLE-ABS-KEY (healthcare AND services) OR TITLE-ABS-KEY (health AND care AND services) OR TITLE-ABS-KEY (health AND services) OR TITLE-ABS-KEY (medical AND care) OR TITLE-ABS-KEY (medical AND services))
3) Acquired immunodeficiency syndrome; AIDS	(TITLE-ABS-KEY (covid-19) OR TITLE-ABS-KEY (covid) OR TITLE-ABS-KEY (coronavirus) OR TITLE-ABS-KEY (corona)) AND (TITLE-ABS-KEY (acquired AND immunodeficiency AND syndrome) OR TITLE-ABS-KEY (aids)) AND (TITLE-ABS-KEY (healthcare AND services) OR TITLE-ABS-KEY (health AND care AND services) OR TITLE-ABS-KEY (health AND services) OR TITLE-ABS-KEY (medical AND care) OR TITLE-ABS-KEY (medical AND services)) AND (LIMIT-TO (DOCTYPE , "ar")) AND (LIMIT-TO (LANGUAGE , "English"))
4) People Living With HIV/AIDS; People Living with HIV; People Living with AIDS; PLHIV	(TITLE-ABS-KEY (covid-19) OR TITLE-ABS-KEY (covid) OR TITLE-ABS-KEY (coronavirus) OR TITLE-ABS-KEY (corona)) AND (TITLE-ABS-KEY (people AND living AND with AND hiv/aids) OR TITLE-ABS-KEY (people AND living AND with AND hiv) OR TITLE-ABS-KEY (people AND living AND with AND aids) OR TITLE-ABS-KEY (plhiv)) AND (TITLE-ABS-KEY (healthcare AND services) OR TITLE-ABS-KEY (health AND care AND services) OR TITLE-ABS-KEY (health AND services) OR TITLE-ABS-KEY (medical AND care) OR TITLE-ABS-KEY (medical AND services)) AND (LIMIT-TO (DOCTYPE , "ar")) AND (LIMIT-TO (LANGUAGE , "English"))
